# Pupillary Light Reflex and Eye Movement Parameters as Objective Measures of Cognitive Decline in Older Adults: A Secondary Analysis of a Multimodal Public Dataset

**DOI:** 10.3390/diagnostics16132102

**Published:** 2026-07-04

**Authors:** Siqi Zhang, Qi Zhao

**Affiliations:** Department of Ophthalmology, The Second Affiliated Hospital of Dalian Medical University, Dalian 116044, China; dr.zhangsiqi@outlook.com

**Keywords:** pupillary light reflex, eye movements, cognitive decline, aging, biomarker, diagnosis

## Abstract

**Background:** Early and objective identification of cognitive decline in aging populations remains a clinical challenge. Pupillary light reflex (PLR) and eye movement parameters represent non-invasive, quantitative biomarkers of autonomic and central nervous system integrity, yet their diagnostic utility for cognitive impairment in community-dwelling older adults, particularly in those with mild or borderline impairment (predominantly GDS-Stage 2), remains underexplored. **Methods:** This cross-sectional study analyzed 383 community-dwelling older adults (mean age 69.78 ± 6.29 years; 68.7% female). Ten PLR parameters (*n* = 202 with complete PLR measurements) and ten eye movement parameters were measured. Associations with cognitive decline (deterioration grade, GDS 2–4) were evaluated using Spearman correlation analysis and multivariate linear regression (adjusted for age, sex, BMI, and hypertension). Stratified analyses and ordinal logistic regression sensitivity analysis were performed to assess robustness. FDR correction (Benjamini–Hochberg) was applied for multiple comparisons. Predictive modeling was conducted using ElasticNet regression with 5-fold cross-validation. **Results:** After FDR correction, resting pupil diameter (ρ = −0.47, q < 0.001), constriction amplitude (ρ = −0.40, q < 0.001), mean constriction velocity (ρ = −0.36, q < 0.001), mean dilation velocity (ρ = −0.36, q < 0.001), and all eye movement velocity parameters (ρ = −0.22 to −0.41, q < 0.001) demonstrated significant negative correlations with cognitive decline. Multivariate regression confirmed resting pupil diameter (β = −0.286, q < 0.001) and constriction amplitude (β = −0.223, q < 0.001) as independent predictors. Sensitivity analysis using ordinal logistic regression yielded consistent results. Predictive modeling yielded modest performance for the primary outcome (PLR-only cross-validated R^2^ = 0.184), whereas models using eye movement features alone or in combination with PLR features performed near chance (R^2^ ≤ 0.04) or showed instability, indicating that these parameters are not yet suitable as standalone diagnostic tools. Exploratory analyses of depression and anxiety were limited by floor effects (≥89% zero scores). **Conclusions:** PLR and eye movement parameters show significant negative associations with cognitive decline in older adults, particularly in a sample skewed toward mild impairment (predominantly GDS-Stage 2). These findings provide preliminary, hypothesis-generating signals that warrant validation in clinical samples with broader cognitive impairment distributions, and these parameters should not yet be considered standalone diagnostic biomarkers.

## 1. Introduction

With the global acceleration of population aging, the prevalence of cognitive decline including mild cognitive impairment and dementia has become a significant and growing public health burden. Epidemiological studies indicate a global prevalence of 5–8% in adults over 60 years, projected to rise substantially in coming decades [1]. Cognitive impairment not only diminishes quality of life and functional independence but is also associated with increased mortality, institutionalization, and caregiver burden [2]. Early and objective identification of cognitive impairment is critical for timely intervention and risk stratification, yet conventional neuropsychological assessments are time-consuming, require specialized personnel, and may be subject to educational and cultural biases [3,4]. Therefore, objective and quantifiable diagnostic biomarkers are urgently needed to complement existing cognitive screening tools in community settings.

Pupillary light reflex (PLR) is an autonomic function jointly regulated by the parasympathetic (Edinger Westphal nucleus) and sympathetic nervous systems. In recent years, portable infrared eye-tracking technology has enabled objective and quantitative measurement of PLR parameters. Previous studies have confirmed characteristic PLR alterations in patients with neurodegenerative diseases, including Alzheimer’s disease and Parkinson’s disease, suggesting its diagnostic utility as a biomarker for neurocognitive disorders [5,6,7]. Notably, the locus coeruleus, a key noradrenergic nucleus involved in attention, arousal, and cognitive resource allocation, provides a shared neurobiological substrate linking PLR to cognitive status. Research demonstrates strong correlations between pupil diameter and locus coeruleus activity, supporting PLR as a non-invasive window into this system’s functional integrity [8,9]. Meanwhile, eye movements, including saccades, fixations, and smooth pursuit, are finely regulated by the central nervous system, with velocity, latency, and accuracy collectively influenced by the frontal cortex, basal ganglia, and cerebellum [10]. Patients with mild cognitive impairment and Alzheimer’s disease exhibit characteristic eye movement alterations, including prolonged saccadic latency, reduced movement velocity, and diminished tracking accuracy [10,11]. Smooth pursuit eye movement, which depends on coordinated activity across the frontal parietal cerebellar circuit and basal ganglia network, reflects visual motor integration capacity and the functional integrity of the central nervous system, making it a promising complementary biomarker for cognitive assessment [12,13,14,15].

No study to date has systematically integrated both PLR and eye movement parameters within a unified analytical framework for assessing cognitive decline in community-dwelling older adults. Given the aforementioned research gaps, this study aims to: (1) systematically evaluate associations between 10 PLR parameters, 10 eye movement parameters, and cognitive decline (operationalized as deterioration grade) in community-dwelling older adults; (2) identify independent predictors of cognitive decline among these ocular parameters; (3) explore the potential utility of these parameters as complementary candidate indicators alongside established cognitive screening tools, while acknowledging the preliminary nature of such associations. We hypothesize that PLR parameters, particularly resting pupil diameter and constriction amplitude, will show significant negative associations with deterioration grade, reflecting autonomic nervous system dysfunction in cognitive decline, and that eye movement parameters will similarly demonstrate significant associations with deterioration grade, reflecting central nervous system involvement.

## 2. Materials and Methods

### 2.1. Research Design and Data Source

This study is a secondary analysis of cross-sectional data from the FPRM dataset, which was originally established as a cross-sectional observational study to systematically characterize pupillary light reflex parameters, smooth pursuit eye movement indicators, and psychological and functional assessment scale scores in older adults [16]. No additional data collection was performed; all data were obtained from the publicly available FPRM dataset.

**Figure 1:**
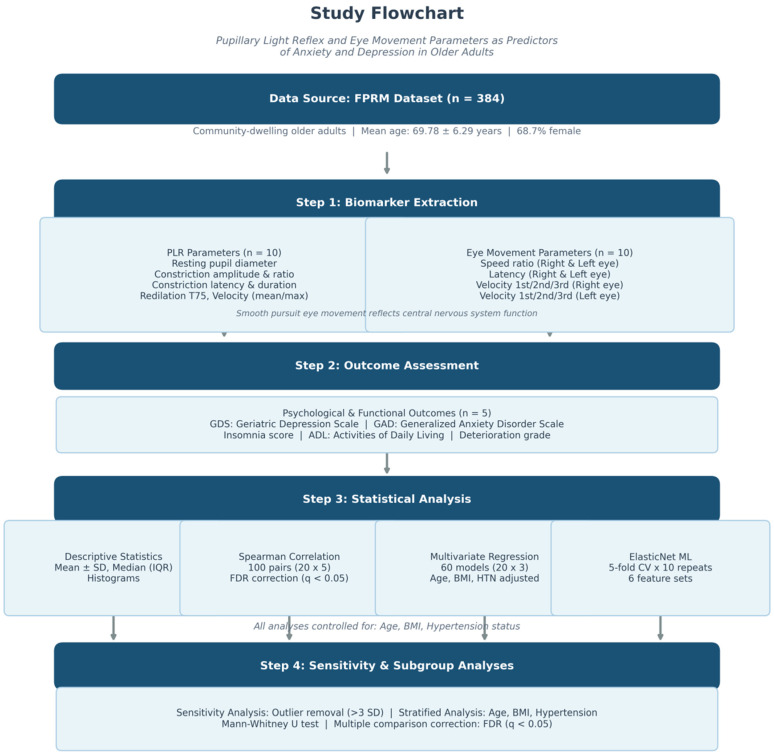
Study flowchart. FPRM, Fundus photography, psychological assessment, retina characteristics, and multimodal imaging; PLR, pupillary light reflex; GDS-15, 15-item Geriatric Depression Scale; GAD, Generalized Anxiety Disorder; ADL, Activities of Daily Living; FDR, false discovery rate; CV, cross-validation; ML, machine learning; BMI, body mass index; HTN, hypertension.

The data acquisition process for this study was as follows: The researchers proactively contacted the corresponding author of the original dataset and obtained data download permissions after signing a formal Data Usage Agreement (DUA). All procedures in this study strictly adhered to the terms specified in the agreement. Because this study involved secondary analysis of de-identified data, no additional ethical review and approval were required. The original study obtained approval from the respective institutional ethics review committee prior to data collection, and all participants provided written informed consent upon enrollment. A flowchart of the study design is shown in Figure 1.

### 2.2. Research Participants

The analytical sample for this study comprised 383 older adult participants (numbered SH1300 to SH1683) from the FPRM dataset. No additional inclusion or exclusion criteria were imposed beyond the original study’s enrollment criteria. All participants underwent standardized pupillary light reflex testing, smooth pursuit eye movement assessment, and a series of psychological and functional evaluation scales. Additionally, the dataset included demographic information including age, sex, height, weight, body mass index (BMI), and self-reported hypertension status.

### 2.3. Research Variables

A total of 31 variables were extracted from the FPRM dataset for analysis, which were organized into the following four categories:

#### 2.3.1. Demographic and Anthropometric Variables

Demographic variables included: participant identification number (ID), sex (male/female), age (years), height (cm), weight (kg), body mass index (BMI, kg/m^2^), and self-reported hypertension status (coded as 1 = hypertension present, 0 = hypertension absent).

#### 2.3.2. Pupillary Light Reflex (PLR) Parameters

A total of 10 PLR parameters were extracted, reflecting the static and dynamic characteristics of autonomic nervous system-mediated pupillary responses. PLR parameters were measured using the Multimodal Eye Functional Imaging and Analysis System (Discovery Model E, SysEye Technology LTD., Chongqing, China), which employs 850 nm laser diode illumination at 82 frames per second for pupillary response measurement, as described in the original FPRM study [16]. All PLR measurements were performed on the right eye only. The following 10 parameters were extracted: Resting pupil diameter: Baseline pupil size prior to light stimulation. Minimum pupil diameter after light stimulation: The smallest pupil diameter recorded following light stimulation. Pupillary constriction amplitude: The absolute reduction from baseline to minimum pupil diameter. Ratio of pupillary constriction amplitude to baseline pupil diameter: The proportion of constriction amplitude relative to the resting diameter. Constriction latency: The time interval between the onset of light stimulation and the initiation of pupillary constriction. Duration of pupillary constriction: The total duration during which the pupil remains in a contracted state. Pupil recovery time (T75): The time required to recover from maximum constriction to 75% of the baseline diameter. Average speed of pupillary constriction: The mean rate of pupil diameter reduction during the constriction phase. Maximum rate of pupillary constriction: The peak rate of pupillary constriction. Average speed of pupillary dilation: The mean rate of pupil diameter recovery during the redilation phase.

#### 2.3.3. Smooth Pursuit Eye Movement Parameters

A total of 10 eye movement parameters were extracted, corresponding to the right and left eyes separately. Eye movements were captured as video recordings using the Multimodal Eye Functional Imaging and Analysis System (Discovery Model E, SysEye Technology LTD., Chongqing, China), as described in the original FPRM study [16]. A threshold-based segmentation approach was used to detect the centers of the pupils and corneal reflection points in each video frame. Eye movements were recorded separately for both the right and left eyes. For each eye (right and left), the following five parameters were extracted:

Ratio of eye movement speed to task target speed: The degree of matching between eye tracking velocity and target motion velocity. Eye motion latency: The time delay between the initiation of target motion and the onset of tracking eye movement. Average velocity of the first eye movement: The mean velocity during the initial tracking phase. Average velocity of the second eye movement: The mean velocity during the second tracking phase. Average velocity of the third eye movement: The mean velocity during the third tracking phase.

Smooth pursuit eye movement is a refined oculomotor function that depends on coordinated activity across the frontal–parietal–cerebellar circuit and basal ganglia network. The quality of this movement reflects visual motor integration capacity and the functional integrity of the central nervous system. The existing literature has reported that smooth pursuit eye movement exhibits reduced gain, prolonged latency, and diminished tracking velocity in the contexts of aging, depression, anxiety disorders, and neurodegenerative diseases. Therefore, in this study, smooth pursuit eye movement parameters were incorporated into the analytical framework as biomarkers reflecting central nervous system functional status, alongside PLR parameters.

#### 2.3.4. Psychological and Functional Assessment Scale Scores

For clarity, in this study, GDS refers to the Global Deterioration Scale (Reisberg et al.) and GDS-15 refers to the 15-item Geriatric Depression Scale. A total of five outcome variables reflecting psychological symptoms and functional status were extracted: 15-item Geriatric Depression Scale (GDS-15) score: A widely used instrument for screening depressive symptoms in older adult populations, with higher scores indicating greater depressive symptom burden [17]. Generalized Anxiety Disorder (GAD) score: A validated assessment tool for anxiety symptom severity, with higher scores reflecting more pronounced anxiety symptoms [18]. Insomnia score: Assessed using the Athens Insomnia Scale (AIS), a validated instrument for evaluating sleep disturbance severity, with higher scores indicating poorer sleep quality [19]. Activities of Daily Living (ADL) score: A scale evaluating functional independence in basic daily activities [20]. Deterioration grade: Cognitive and functional status was assessed using the Global Deterioration Scale (GDS), as documented in the original FPRM dataset [16]. The GDS was originally developed and validated by Reisberg et al. [21] and is one of the most widely used instruments for staging the severity of cognitive decline in older adults. The GDS classifies cognitive and functional status into seven stages (GDS 1–7), where higher grades indicate more severe impairment, with each stage defined by detailed descriptors of cognitive symptoms and functional abilities. In the FPRM dataset, participants were classified into GDS stages by trained clinicians based on comprehensive neuropsychological assessments. Three GDS grades were present in our analytical sample: Grade 2 = normal aging with subtle cognitive complaints (GDS Stage 2); Grade 3 = mild cognitive decline (GDS Stage 3, early forgetfulness exceeding normal aging, with objective findings on testing but preserved daily function); Grade 4 = moderate cognitive decline (GDS Stage 4, mild cognitive impairment with clear-cut deficits). The GDS is strongly correlated with established cognitive screening tools, including the Mini-Mental State Examination (MMSE) and Montreal Cognitive Assessment (MoCA), where higher GDS stages correspond to progressively lower scores on these instruments. Several variables known to influence PLR and eye movement parameters, including ocular disease, diabetes, neurological disease, medication use, and autonomic dysfunction, were not collected in the FPRM dataset and therefore could not be adjusted for in the present analysis.

### 2.4. Data Preprocessing

All data preprocessing and analysis were performed using Python 3.14. The specific preprocessing steps are as follows:

#### 2.4.1. Variable Name Standardization and Recoding

Original variable names from the FPRM dataset were mapped to standardized short variable names to facilitate programmatic analysis. The sex variable was recoded as a binary numeric variable (male = 1, female = 0). All continuous variables were converted to numeric format, and non-numeric data entries were treated as missing values.

#### 2.4.2. Outlier Treatment

To mitigate the influence of extreme values on analytical results, all continuous PLR indicators and smooth pursuit eye movement indicators were winsorized at the 1st and 99th percentiles. This method effectively constrains the potential impact of measurement errors or data entry mistakes on analytical results while preserving the ranking of observations.

#### 2.4.3. Missing Data Handling

Missing data in this study were attributable to the differential measurement protocol of the original FPRM dataset, rather than participant dropout or loss to follow-up. Specifically, PLR parameters were only measured in participants who underwent the full pupillary response testing protocol (*n* = 202), while eye movement parameters were measured in a larger subset (*n* > 300). Participants with complete data for both PLR and eye movement parameters were included in the combined analyses. Complete-case analysis was employed for each specific analysis, meaning only participants with no missing values across all variables involved in each model were retained. The proportion of missing data for each variable is reported in the descriptive statistics table (Supplementary Table S1).

### 2.5. Statistical Analysis

Statistical analysis was conducted in the following five sequential steps:

#### 2.5.1. Descriptive Statistics

Descriptive statistics (mean ± SD, median, IQR) were calculated for all variables.

#### 2.5.2. Correlation Analysis

Spearman rank correlation coefficients were used to evaluate bivariate associations in the following two groups: (1) Associations between PLR parameters and cognitive decline (deterioration grade): 10 PLR parameters × 1 primary outcome; (2) Associations between smooth pursuit eye movement parameters and cognitive decline: 10 eye movement parameters × 1 primary outcome. Given the primary focus on cognitive decline as the main diagnostic outcome of interest, and the exploratory nature of the secondary mental health analyses (due to floor effects in GDS-15 and GAD scores), the primary FDR correction was applied across the 20 biomarker–cognitive decline pairs. Associations with GDS-15, GAD, insomnia, and ADL are reported as exploratory uncorrected findings. Spearman correlation was selected because several variables exhibited non-normal distributions.

#### 2.5.3. Multivariate Linear Regression Analysis

To evaluate whether the associations between PLR/eye movement parameters and cognitive decline were independent of potential confounding factors, separate multivariate linear regression models were constructed for each biomarker–deterioration grade pair. Given that deterioration grade is an ordinal variable (GDS 2, 3, 4), linear regression was employed as the primary analytical approach for the following reasons: (1) standardized coefficients permit direct comparison of effect sizes across different biomarkers; (2) the sample size (*n* = 383) is sufficient to support approximate normality of residuals in our models; (3) this approach has been used in published studies with ordinal cognitive outcomes. As a sensitivity analysis, ordinal logistic regression (proportional odds model) was also performed.

#### 2.5.4. Predictive Modeling

To evaluate the overall predictive capability of PLR and eye movement parameters for cognitive decline and to assess their relative contributions, ElasticNet regularized regression models were employed, with training and evaluation conducted through repeated 5-fold cross-validation (5 folds × 10 repeats, yielding a total of 50 model evaluations). Three feature sets were evaluated: PLR parameters only, eye movement parameters only, and the combined feature set. For each outcome, cross-validated R^2^ was reported as the primary metric of predictive performance.

#### 2.5.5. Stratified and Sensitivity Analyses

Stratified analyses: To evaluate whether the observed associations were modified by demographic or health characteristics, participants were stratified into two groups each for age (median-split), sex (male/female), BMI (WHO classification: <25 vs. ≥25), and hypertension status (present/absent). Within each stratum, Spearman correlation coefficients between the primary PLR/eye movement parameters and deterioration grade were calculated separately. The Mann Whitney U test was used to compare differences in PLR and eye movement parameters between subgroups, with FDR correction applied across comparisons. Results were visualized as stratified box plots. Sensitivity analyses: Two sensitivity analyses were performed: (1) After excluding statistical outliers identified using the IQR rule (values below Q1 − 1.5 × IQR or above Q3 + 1.5 × IQR), Spearman correlation coefficients were recalculated to evaluate the influence of extreme values; (2) Ordinal logistic regression (proportional odds model) was performed for primary biomarker–deterioration grade associations to evaluate robustness to the ordinal nature of the outcome variable.

#### 2.5.6. Sensitivity Analysis Using Ordinal Logistic Regression

To evaluate the robustness of findings to the ordinal nature of the deterioration grade outcome, sensitivity analyses were conducted using ordinal logistic regression (proportional odds model) for the primary biomarker–deterioration grade associations. Results were considered consistent with the primary linear regression findings if the direction and statistical significance of associations were preserved.

### 2.6. Software and Reproducibility

All analyses were implemented using Python 3.14 and the following open-source packages: pandas (3.0.2), NumPy (2.4.4), SciPy (1.17.1), statsmodels (0.14.6), scikit-learn (1.8.0), matplotlib (3.10.8), and seaborn (0.13.2). The analysis scripts were organized in a modular workflow comprising a main control script, a table module, and a figure module. All analysis code, parameter settings, and random seeds used in ElasticNet cross-validation have been documented and are available in the Supplementary Materials (Code S1). The complete analysis pipeline, including data preprocessing, statistical analyses, and visualization, is organized in a modular workflow with comprehensive inline documentation to ensure full reproducibility of results. Two-sided tests were employed throughout, with *p* < 0.05 (or FDR-corrected q < 0.05) used as the threshold for statistical significance.

### 2.7. Sample Size and Statistical Power

This study did not perform an a priori sample size calculation, as it was a secondary analysis of an existing dataset. The sample size was determined by the original FPRM study design (*n* = 384 for multimodal imaging subset; *n* = 202 for PLR parameters). Post hoc power analysis indicates that with *n* = 383 and α = 0.05, the study had >80% power to detect correlations of r ≥ 0.15, corresponding to a small-to-medium effect size.

### 2.8. Ethical Statement

This study is a secondary analysis of de-identified data from the FPRM dataset [16]. As the original study received ethical approval and participants provided informed consent, and as this study involved only analysis of existing de-identified data, no additional ethics review was required. Data access was granted under a formal Data Usage Agreement.

## 3. Result

### 3.1. Demographic and Cognitive/Functional Descriptive Statistics

**Figure 2:**
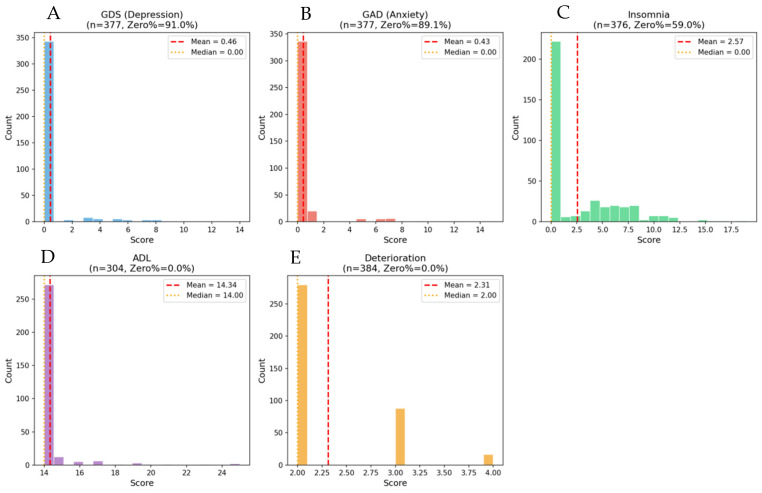
Distributions of cognitive and functional assessment scores in the study population. Histograms showing score distributions for (**A**) GDS-15 (15-item Geriatric Depression Scale), (**B**) GAD (Generalized Anxiety Disorder), (**C**) Insomnia score, (**D**) ADL (Activities of Daily Living), and (**E**) Deterioration grade. Colored bars represent frequencies, with different colors used for each subplot. Dashed red lines mark means; dotted orange lines mark medians.

This study enrolled 383 elderly participants (SH1300 to SH1683), including 263 females (68.7%) and 120 males, with a mean age of 69.78 ± 6.29 years. Of these, 202 participants had complete PLR measurements, while 181 had only eye movement data; no significant baseline differences were observed between these subgroups (Supplementary Table S1). Table 1 presents the detailed baseline characteristics. The distributions of cognitive and functional assessment scores are illustrated in Figure 2. The deterioration grade showed a spread distribution (grade 2: *n* = 280, 72.9%; grade 3: *n* = 88, 22.9%; grade 4: *n* = 16, 4.2%), indicating sufficient variability for correlation analysis. In contrast, GDS-15 and GAD scores exhibited pronounced floor effects characteristic of community-dwelling samples, with ≥89% zero scores.

### 3.2. Correlation Analysis: Primary Findings on Cognitive Decline

**Figure 3:**
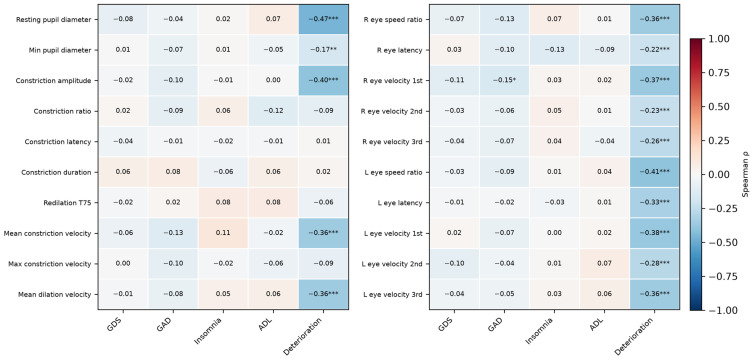
Spearman correlation heatmap between pupillary light reflex parameters, eye movement parameters, and psychological/functional scores. Correlation heatmap of PLR parameters, eye movement parameters, and cognitive/functional outcomes. PLR, pupillary light reflex; Eye, smooth pursuit eye movement; GDS-15, 15-item Geriatric Depression Scale; GAD, Generalized Anxiety Disorder; ADL, Activities of Daily Living; Deterioration, cognitive deterioration grade. Color intensity reflects Spearman correlation strength; asterisks indicate FDR-corrected significance (* q < 0.05, ** q < 0.01, *** q < 0.001). Significant negative correlations are observed between both PLR/eye movement parameters and cognitive deterioration grade, while associations with GDS-15, GAD, and ADL are generally weak.

We first examined the associations between PLR/eye movement parameters and cognitive decline (deterioration grade) as the primary diagnostic outcome of interest (Table 2, Figure 3). After FDR correction, deterioration grade showed significant negative correlations with multiple PLR parameters, including resting pupil diameter (ρ = −0.47, q < 0.001), constriction amplitude (ρ = −0.40, q < 0.001), mean constriction velocity (ρ = −0.36, q < 0.001), and mean dilation velocity (ρ = −0.36, q < 0.001). Similarly, deterioration grade showed significant negative correlations with all eye movement velocity parameters (ρ = −0.22 to −0.41, q < 0.001), indicating that greater cognitive decline was associated with markedly diminished pupillary light reflex and oculomotor performance.

### 3.3. Multivariate Regression Analysis: Cognitive Decline

**Figure 4:**
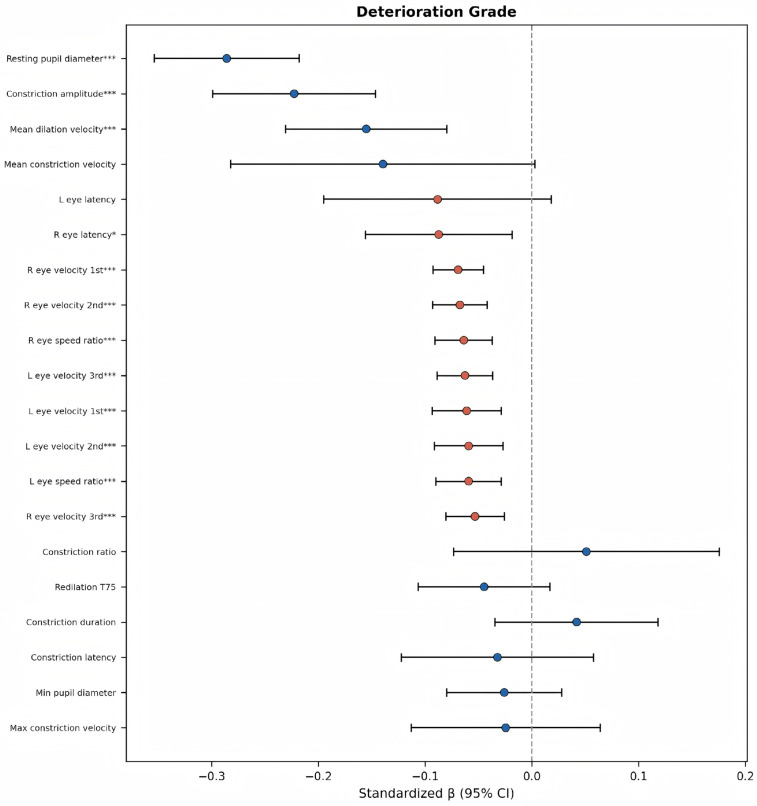
Multivariable regression: PLR & Eye movement Metrics. Forest plot of standardized regression coefficients (β) for the associations between biomarkers and deterioration grade. Blue indicates PLR parameters; red indicates eye movement parameters. Horizontal lines represent 95% confidence intervals (CIs). The vertical dashed line indicates the null effect line (β = 0), and confidence intervals not crossing the null line indicate statistical significance (* *p* < 0.05, *** *p* < 0.001). All models were adjusted for age, sex, BMI, and hypertension.

We constructed multivariate linear regression models with age, sex, BMI, and hypertension as control variables to examine the predictive effects of each biomarker on cognitive deterioration grade. As illustrated in Figure 4: Multivariate regression analysis confirmed that resting pupil diameter (β = −0.286, q < 0.001) and constriction amplitude (β = −0.223, q < 0.001) remained significant independent predictors of cognitive decline after adjustment for age, sex, BMI, and hypertension (Table 3). Among eye movement parameters, the velocity ratio of the left eye (β = −0.28, q < 0.001) and the first saccade velocity of both eyes (β = −0.24 to −0.27, q < 0.001) emerged as the strongest independent predictors, remaining significant after covariate adjustment.

Sensitivity analysis using ordinal logistic regression (proportional odds model) confirmed the robustness of our findings. After FDR correction, resting pupil diameter (OR = 0.354, 95% CI: 0.27–0.47, *p* < 0.001), constriction amplitude (OR = 0.12, 95% CI: 0.06–0.24, *p* < 0.001), constriction velocity (OR = 0.502, 95% CI: 0.35–0.72, *p* < 0.001), and redilation velocity (OR = 0.026, 95% CI: 0.00–0.14, *p* < 0.001) remained significantly associated with higher deterioration grade. (Supplementary Table S2).

### 3.4. Exploratory Analyses: Depression, Anxiety, and Other Outcomes

In exploratory analyses (uncorrected for multiple comparisons due to the limited statistical power in these domains), the associations between PLR/eye movement parameters and depression (GDS-15), anxiety (GAD), insomnia, and ADL scores were generally weak and non-significant after FDR correction. Importantly, this is consistent with the pronounced floor effects observed in GDS-15 and GAD scores (≥89% zero scores) in this community-dwelling sample, which substantially limits statistical power to detect associations in these domains. These findings should be interpreted as exploratory and hypothesis-generating rather than definitive null results. Future studies in clinical samples with broader symptom ranges are needed to definitively evaluate associations between oculomotor biomarkers and mental health outcomes.

### 3.5. Predictive Modeling Results

ElasticNet regression models were constructed to predict three outcome measures (Deterioration grade, GDS-15 score, and GAD score), with performance evaluated across three feature sets (PLR only, Eye only, and PLR + Eye combined) using 50 iterations of 5-fold cross-validation (5 folds × 10 repeats).

For the primary outcome (Deterioration grade), pupillary light reflex parameters (PLR only) achieved the best predictive performance, with R^2^ = 0.184 ± 0.142, indicating that PLR features explained approximately 18.4% of the variance in cognitive deterioration grade. Eye movement parameters alone showed negligible predictive ability (R^2^ = −0.020 ± 0.047). Interestingly, the combined feature set (PLR + Eye) yielded inferior results (R^2^ = 0.040 ± 0.391), likely attributable to the reduced sample size (*n* = 147) and the substantially larger standard deviation reflecting increased model instability with a larger feature space relative to the effective sample size.

For the exploratory outcomes, both GDS-15 and GAD scores demonstrated negative R^2^ values across all three feature sets. For GDS-15, results were: PLR only (R^2^ = −0.072 ± 0.115), Eye only (R^2^ = −0.051 ± 0.058), and PLR + Eye (R^2^ = −0.095 ± 0.182). For GAD, results were: PLR only (R^2^ = −0.436 ± 1.038), Eye only (R^2^ = −0.172 ± 0.616), and PLR + Eye (R^2^ = −1.192 ± 4.235). These consistently negative R^2^ values indicate that the ElasticNet models performed worse than simply using the sample mean as the predictor, suggesting that none of the feature sets contain sufficient information to predict GDS-15 or GAD scores in a linear framework. This finding is consistent with the distribution characteristics of both scales: ≥89% of participants scored 0 on the GDS-15, and ≥89% scored 0 on the GAD, representing a pronounced floor effect that severely limits the ability of linear regression models to capture meaningful variance. Notably, the GAD scale exhibited a more severe distribution skew than the GDS-15, as evidenced by its larger negative R^2^ values and dramatically inflated standard deviations, particularly in the combined feature set. Detailed results are presented in Table 4.

### 3.6. Stratified and Sensitivity Analyses

Stratified analyses by age, sex, BMI, and hypertension status confirmed that the significant associations between PLR/eye movement parameters and cognitive decline were generally consistent across demographic subgroups. Sensitivity analyses excluding statistical outliers (IQR-based) further confirmed the robustness of the primary findings.

**Figure 5:**
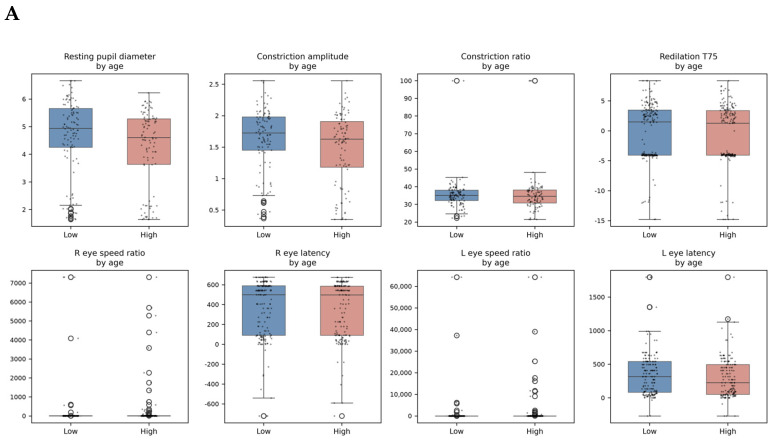

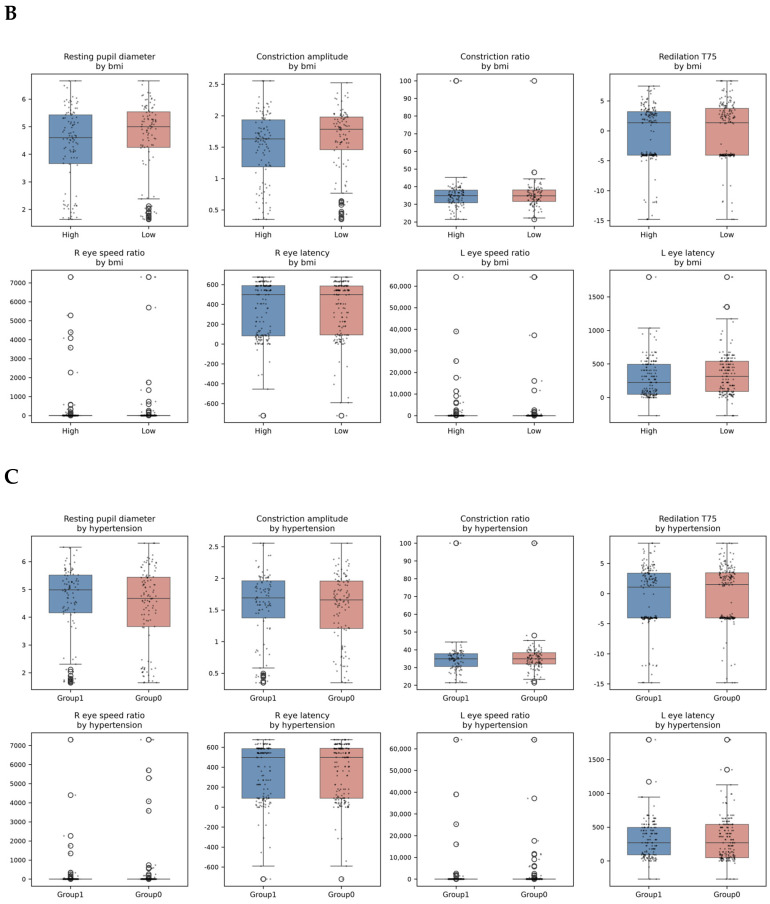
Distribution of Biomarkers across Subgroups Stratified by Age, BMI, and Hypertension Status. Stratified analyses of biometric parameters by (**A**) age group, (**B**) BMI group, and (**C**) hypertension status. Blue boxes represent the high-risk group (older age, higher BMI, or hypertensive); red boxes represent the low-risk group (younger age, lower BMI, or normotensive). Box boundaries indicate interquartile range (IQR); horizontal line inside box marks median; whiskers extend to 1.5 × IQR; open circles represent outliers. PLR, pupillary light reflex parameters; Eye Movement, ocular motor parameters; BMI, body mass index.

We conducted stratified analyses by age, BMI, and hypertension status, respectively. As illustrated in Figure 5, among the three stratified analyses, the majority of biomarkers showed no significant differences across subgroups (Mann Whitney U test; all FDR-corrected q > 0.05). Although weak trends were observed in certain indicators (e.g., shorter constriction duration in the hypertension group, smaller pupil diameter in the older age group), none reached statistical significance after multiple comparison correction, suggesting that age, BMI, and hypertension are not significant effect modifiers of the main associations in this study, and the influence of covariates on the relationships between pupillary and eye movement parameters and mental health outcomes is limited.

## 4. Discussion

This study systematically evaluated the diagnostic utility of PLR and eye movement parameters for identifying cognitive decline in 383 community-dwelling older adults. The results demonstrated significant negative correlations between PLR parameters (ρ = −0.36 to −0.47, q < 0.001) and eye movement parameters (ρ = −0.22 to −0.41, q < 0.001) with cognitive decline, with resting pupil diameter and constriction amplitude emerging as independent predictors. These findings suggest that PLR and eye movement parameters may serve as complementary candidate indicators for cognitive decline, providing an objective tool to complement conventional neuropsychological testing in community settings. Further validation in clinical samples with broader cognitive impairment distributions is warranted.

The most robust finding of this study was the significant negative correlation between PLR parameters and cognitive decline. From a neuroanatomical perspective, the pupillary light reflex is primarily regulated by the Edinger–Westphal nucleus (parasympathetic) and the sympathetic hypothalamic–spinal pathway. The locus coeruleus, the primary source of noradrenergic innervation, serves as a critical node linking autonomic regulation, attention, and cognitive resource allocation [22]. Research has demonstrated that dysfunction in the locus coeruleus–norepinephrine system is closely associated with the development of cognitive disorders, including Alzheimer’s disease [23]. Notably, there is strong evidence that pupillary diameter is correlated with locus coeruleus activity, suggesting that PLR measurements may provide a non-invasive diagnostic window into this system’s functional status [22,23]. Dawidziuk et al. confirmed in a systematic review that PLR parameters demonstrate characteristic alterations in patients with neurodegenerative diseases including Parkinson’s disease [6]. Castellotti et al. proposed that pupillary diameter can serve as a biomarker of cognitive function, providing an objective basis for cognitive screening [11]. Consistent with these prior findings, our results identified resting pupil diameter and constriction amplitude as the most potent PLR predictors of cognitive decline, which may reflect impaired autonomic regulatory capacity and reduced iris musculature function associated with neurodegeneration.

Eye movement velocity parameters were also significantly negatively correlated with cognitive decline (ρ = −0.22 to −0.41, q < 0.001). Smooth pursuit eye movement depends on coordinated activity across the frontal–parietal–cerebellar circuit and basal ganglia network, and its quality reflects visual–motor integration capacity and the functional integrity of the central nervous system [24]. Opwonya et al. reported that patients with mild cognitive impairment and Alzheimer’s disease exhibit characteristic alterations in saccadic eye movements, including prolonged saccadic latency and reduced movement velocity [12]. Anagnostou et al. further noted that eye movement abnormalities are promising biomarkers for neurodegenerative dementias [25]. The significant associations observed in this study are consistent with these prior findings and suggest that eye movement parameters hold promise as complementary objective indicators for neurocognitive assessment.

The relatively strong associations of PLR and eye movement parameters with cognitive decline may be related to the shared neural circuits they depend on. The PLR primarily relies on parasympathetic nuclei at the midbrain level, while smooth pursuit eye movement involves the integrity of the cortical–basal ganglia–cerebellar circuit. Cognitive decline, particularly impairment in cognitive domains involving attention, executive function, and motor coordination, may simultaneously affect these two physiological systems that are independent yet share some neural resources [26,27].

In exploratory analyses, the associations between PLR/eye movement parameters and depression (GDS-15) and anxiety (GAD) scores were generally weak. It is important to recognize that this community-dwelling sample exhibited pronounced floor effects in both GDS-15 and GAD scores (≥89% zero scores), reflecting the relatively favorable mental health status of the participants. This statistical limitation substantially reduces power to detect associations in these domains. Prior studies have reported PLR alterations in clinically diagnosed depression [28] and associations between eye movement abnormalities and depressive symptoms [29,30]; however, these findings derive from clinical populations with substantially higher symptom burden. The present null findings for depression and anxiety should be interpreted as reflecting sample characteristics rather than evidence against biological associations. Future studies should validate these associations in clinical samples with broader symptom ranges.

This study has several strengths. First, this is the first study to systematically integrate both PLR and eye movement parameters within a unified analytical framework in relation to cognitive decline in community-dwelling older adults. Second, to ensure the robustness and transparency of our findings, we employed a comprehensive analytical strategy encompassing multiple complementary approaches: (1) Spearman correlation analysis for bivariate associations; (2) multivariate linear regression with covariate adjustment; (3) ElasticNet regularized regression for predictive modeling; (4) stratified analyses to examine potential effect modifiers; (5) ordinal logistic regression sensitivity analysis to validate the appropriateness of treating deterioration grade as a continuous outcome. Ordinal logistic regression sensitivity analysis confirmed the robustness of our findings, with resting pupil diameter (OR = 0.354, 95% CI: 0.27–0.47, *p* < 0.001), constriction amplitude (OR = 0.12, 95% CI: 0.06–0.24, *p* < 0.001), and constriction velocity (OR = 0.502, 95% CI: 0.35–0.72, *p* < 0.001) remaining significantly associated with higher deterioration grade after FDR correction. Stratified analyses demonstrated consistent associations across demographic subgroups (age, sex, BMI, hypertension), supporting the generalizability of our findings within this population. Third, the application of FDR correction for multiple comparisons, combined with robust regression methods, strengthens the validity of our findings.

This study has several limitations that should be carefully considered when interpreting the findings. The cross-sectional design precludes causal inference, and future longitudinal studies are warranted. The community-dwelling sample exhibited a skewed cognitive status distribution (predominantly GDS Grade 2), which limited statistical power in severe cognitive decline groups and restricts generalizability to clinical populations. Although the GDS is a validated instrument, the raw neuropsychological test scores (e.g., MMSE, MoCA) were not available in the FPRM dataset for independent validation. Residual confounding cannot be excluded, as numerous factors, including ocular disease, diabetes, neurological disease, medication use, and autonomic dysfunction, may influence PLR and eye movement parameters but were not collected. The predictive performance of the ElasticNet models was modest (cross-validated R^2^ = 0.184 for PLR-only), indicating that these parameters cannot yet be considered standalone diagnostic biomarkers and should be interpreted as complementary candidate indicators. Missing data were attributable to the differential measurement protocol (PLR n = 202 vs. eye movement n > 300), which may introduce selection bias. In particular, the absence of ophthalmic and neurological covariates in the FPRM dataset precludes adjustment for factors that are known to influence oculomotor function.

## 5. Conclusions

This study systematically evaluated the associations between PLR and eye movement parameters with cognitive decline in 383 community-dwelling older adults. The results demonstrated significant negative correlations between PLR parameters (ρ = −0.36 to −0.47) and eye movement parameters (ρ = −0.22 to −0.41) with cognitive decline, with resting pupil diameter and constriction amplitude emerging as independent predictors. Derived from a sample skewed toward mild cognitive impairment (predominantly GDS-Stage 2), these findings suggest that these ocular parameters may serve as complementary candidate indicators for cognitive decline, although they cannot be considered standalone biomarkers for cognitive function assessment in older adults yet. Exploratory analyses of depression and anxiety were limited by floor effects in this community sample, and definitive conclusions regarding those associations require validation in clinical populations with broader symptom ranges. Future longitudinal studies in clinical samples with diverse cognitive profiles are warranted.

## Figures and Tables

**Table 1:** Comparison of baseline characteristics of the research subjects.

Variable	Mean ± SD	Median (IQR)	Missing (%)
Age (years)	69.78 ± 6.29	69.00 (66.00–74.00)	0.0
Sex (male, *n* (%))	120 (31.3%)	—	0.0
Height (cm)	160.71 ± 7.05	160.00 (156.00–165.00)	0.0
Weight (kg)	61.03 ± 9.06	60.00 (55.00–65.00)	0.0
BMI (kg/m^2^)	23.61 ± 3.09	23.03 (21.63–24.98)	0.0
Hypertension (*n* (%))	182 (47.4%)	—	0.0
GDS-15 score	0.46 ± 1.66	0.00 (0.00–0.00)	1.8
GAD score	0.43 ± 1.68	0.00 (0.00–0.00)	1.8
Insomnia score	2.57 ± 3.72	0.00 (0.00–5.00)	2.1
ADL score	14.34 ± 1.35	14.00 (14.00–14.00)	20.8
Deterioration grade	2.31 ± 0.55	2.00 (2.00–3.00)	0.0

Continuous variables are described as mean ± SD and median (IQR); categorical variables are reported as frequency and percentage (*n* [%]). Abbreviations: BMI, body mass index; GDS-15, 15-item Geriatric Depression Scale; GAD, Generalized Anxiety Disorder Scale; ADL, Activities of Daily Living Scale; IQR, interquartile range.

**Table 2:** Spearman correlation coefficients between PLR/eye movement parameters and deterioration grade (primary outcome).

Bio_var	Category	Psych_var	Spearman_rho	*p*	n	*q* (FDR)	Significance
Resting pupil diameter	PLR	deterioration	−0.468	0.000	202	0.000	***
Minimum pupil diameter	PLR	deterioration	−0.167	0.001	367	0.009	**
Constriction amplitude	PLR	deterioration	−0.402	0.000	202	0.000	***
Constriction ratio	PLR	deterioration	−0.087	0.217	202	0.571	
Constriction latenc	PLR	deterioration	0.013	0.809	367	0.927	
Constriction duration	PLR	deterioration	0.024	0.646	367	0.911	
Recovery T75	PLR	deterioration	−0.058	0.266	366	0.620	
Constriction velocity (avg)	PLR	deterioration	−0.356	0.000	202	0.000	***
Constriction velocity (max)	PLR	deterioration	−0.085	0.103	366	0.422	
Dilation velocity (avg)	PLR	deterioration	−0.359	0.000	202	0.000	***
Right eye velocity ratio	Eye Movement	deterioration	−0.358	0.000	316	0.000	***
Right eye latency	Eye Movement	deterioration	−0.217	0.000	316	0.001	***
Right eye velocity (1st)	Eye Movement	deterioration	−0.373	0.000	316	0.000	***
Right eye velocity (2nd)	Eye Movement	deterioration	−0.230	0.000	316	0.000	***
Right eye velocity (3rd)	Eye Movement	deterioration	−0.264	0.000	316	0.000	***
Left eye velocity ratio	Eye Movement	deterioration	−0.406	0.000	314	0.000	***
Left eye latency	Eye Movement	deterioration	−0.328	0.000	314	0.000	***
Left eye velocity (1st)	Eye Movement	deterioration	−0.381	0.000	314	0.000	***
Left eye velocity (2nd)	Eye Movement	deterioration	−0.276	0.000	314	0.000	***
Left eye velocity (3rd)	Eye Movement	deterioration	−0.357	0.000	313	0.000	***

Abbreviations: PLR, pupillary light reflex; Eye, smooth pursuit eye movement; FDR, false discovery rate (Benjamini Hochberg). ** *p* < 0.01, *** *p* < 0.001.

**Table 3:** Multivariate regression: PLR and eye movement parameters predicting cognitive decline (deterioration grade), adjusted for age, sex, BMI, and hypertension.

Biomarker	Category	β	95% CI	*p*	q (FDR)	*n*
Resting pupil diameter	PLR	−0.286	(−0.38, −0.19)	<0.001	<0.001	195
Constriction amplitude	PLR	−0.223	(−0.32, −0.13)	<0.001	<0.001	195
Constriction velocity (avg)	PLR	−0.178	(−0.27, −0.08)	<0.001	0.003	195
Dilation velocity (avg)	PLR	−0.189	(−0.29, −0.09)	<0.001	0.002	195
Left eye velocity ratio	Eye Movement	−0.283	(−0.38, −0.19)	<0.001	<0.001	303
Left eye velocity (1st)	Eye Movement	−0.268	(−0.36, −0.17)	<0.001	<0.001	303
Right eye velocity (1st)	Eye Movement	−0.244	(−0.34, −0.15)	<0.001	<0.001	305
Right eye velocity ratio	Eye Movement	−0.227	(−0.32, −0.13)	<0.001	<0.001	305

Only biomarkers with q (FDR) < 0.05 are shown. β = standardized regression coefficient; CI = confidence interval; FDR = false discovery rate (Benjamini Hochberg). Covariates in all models: age, sex, BMI, hypertension.

**Table 4:** Predictive Performance of ElasticNet Models for Deterioration Grade, GDS-15, and GAD Prediction.

Outcome	Feature Set	R^2^ (mean ± SD)	r2_mean	r2_sd	*n*	n_features
Deterioration	PLR only	0.184 ± 0.142	0.184	0.142	202	10
Deterioration	Eye only	−0.020 ± 0.047	−0.020	0.047	281	10
Deterioration	PLR + Eye	0.040 ± 0.391	0.040	0.391	147	20
GDS-15	PLR only	−0.072 ± 0.115	−0.072	0.115	197	10
GDS-15	Eye only	−0.051 ± 0.058	−0.051	0.058	275	10
GDS-15	PLR + Eye	−0.095 ± 0.182	−0.095	0.182	143	20
GAD	PLR only	−0.436 ± 1.038	−0.436	1.038	197	10
GAD	Eye only	−0.172 ± 0.616	−0.172	0.616	275	10
GAD	PLR + Eye	−1.192 ± 4.235	−1.192	4.235	143	20

Predictive Performance of ElasticNet Models for Deterioration Grade, GDS-15, and GAD Prediction. Data are presented as mean ± SD across 50 evaluation iterations (5-fold cross-validation × 10 repeats). R^2^ indicates the proportion of variance explained by the model. PLR = pupillary light reflex parameters; Eye = eye movement parameters; GDS-15 = 15-item Geriatric Depression Scale; GAD = Generalized Anxiety Disorder scale; n = number of participants with complete data for the respective feature set; n_features = number of features included in the model.

## Data Availability

The data analyzed in this study were obtained from the publicly available Functional Pupillary Response Measurement (FPRM) dataset [16]. Access to the original data requires contacting the dataset authors for permission and signing a formal Data Usage Agreement.

## Supplementary Materials

The following supporting information can be downloaded at: https://www.mdpi.com/article/10.3390/diagnostics16132102/s1, Table S1: Baseline Characteristics Comparison between PLR-Available and PLR-Missing Groups. Table S2: Ordinal Logistic Regression Analysis-Pupil Parameters and Deterioration Grade. Code S1.
